# Risk factors for poor tuberculosis treatment outcome in Finland: a cohort study

**DOI:** 10.1186/1471-2458-7-291

**Published:** 2007-10-14

**Authors:** Tuula Vasankari, Pekka Holmström, Jukka Ollgren, Kari Liippo, Maarit Kokki, Petri Ruutu

**Affiliations:** 1 Department of Infectious Disease Epidemiology, National Public Health Institute, Mannerheimintie 166, 00300 Helsinki, Finland; 2 Department of Respiratory Medicine, Turku University Hospital, Paimio Hospital, Alvar Aallon tie 275, 21540 Preitilä, Finland

## Abstract

**Background:**

We investigated the patient- and treatment-system dependent factors affecting treatment outcome in a two-year cohort of all treated culture-verified pulmonary tuberculosis (TB) cases to establish a basis for improving outcomes.

**Methods:**

Medical records of all cases in 1995 – 1996 were abstracted to assess outcome of treatment. Outcome was divided into three groups: favourable, death and other unfavourable. Predictors of unfavourable outcome were assessed in univariate and multivariate analysis.

**Results:**

Among 629 cases a favourable outcome was achieved in 441 (70.1%), 17.2% (108) died and other unfavourable outcome took place in 12.7% (80). Significant independent risk factors for death were male sex, high age, non-HIV -related immunosuppression and any other than a pulmonary specialty being responsible for stopping treatment. History of previous tuberculosis was inversely associated with the risk of death. For other unfavourable treatment outcomes, significant risk factors were pause(s) in treatment, treatment with INH+RIF+EMB/SM, and internal medicine specialty being responsible at the end of the treatment.

**Conclusion:**

We observed a significant association with unfavourable outcome for the specialty responsible for treatment being other than pulmonary, but not for the volume of cases, which has implications for system arrangements. Poor outcomes associated with immunosuppression and advanced age, with frequent comorbidity, stress a low threshold of suspicion, availability of rapid diagnostics, and early empiric treatment as probable approaches in attempting to improve treatment outcomes in countries with very low incidence of TB.

## Background

Early diagnosis of tuberculosis and effective treatment are the key elements in reduction of transmission of infection and finally achieving elimination of TB [1]. World Health Organization (WHO) has set the international target value for a favourable treatment outcome at 85% [2]. Treatment outcome monitoring is a core part of surveillance necessary to succeed in tuberculosis elimination [3]. The WHO has published a recommendations for assessing the outcome of tuberculosis treatment in 1990's [4], revised recently [5,6].

In many industrialized countries with good treatment facilities and a secured supply of drugs free of charge for patients, treatment results have not reached the targets set by WHO [7-18]. The main reason for this is the high rate of death as an unfavourable outcome, frequently with much comorbidity from other diseases. Incomplete treatment carries a risk of development of resistance, increased disease transmission, and increased morbidity and mortality [19]. In our earlier report from Finland, a favourable outcome was reached in only 65%, death being the outcome in as much as 19%, and defaulting, transferring out or physician's decision to stop treatment early being reasons for other unfavourable outcomes in 12% of the cases [20].

The specific reasons for unsuccessful outcomes are important in order to improve treatment systems. In a recent outcome analysis from Norway, where the large majority of TB cases are in immigrants, only high age and isoniazid (INH) resistance were significant risk factors for non-successful outcome [21]. In earlier studies, high age, alcoholism, HIV-infection, male sex and immigration have been associated with unfavourable outcomes [7,12,22].

In low incidence countries, many of the clinical units treating tuberculosis patients have small and decreasing numbers of patients. This necessitates an assessment of the need to concentrate patients in fewer units to retain the level of experience sufficient for successful outcomes. There is varying evidence from assessments made in other areas of complex treatments, such as leukaemia, AIDS, demanding surgery and myocardial infarction, that the volume of treatments has an effect on the outcome [23,24]. It is possible that treatment organisation and implementation in bigger hospitals are more effective and up to date [25]. However, there is little data available on whether the volume of tuberculosis patients treated would associate with favourable outcomes.

We assessed the patient and treatment system dependent factors affecting treatment outcome in a national, population-based two-year cohort of all culture-verified pulmonary tuberculosis cases in Finland, with a TB incidence of 12,5 per 100 000 per year during the study period, to establish a basis for improving the proportion of favourable outcomes.

## Methods

### Study cohort, case definitions and data collection

The method of identifying all culture-confirmed tuberculosis cases in Finland, with the first positive culture sample date between January 1^st^, 1995, to December 31^st^, 1996 (N = 1059), present in either the National Infectious Disease Register (NIDR) or through a separate query to all microbiological laboratories has been described elsewhere [26].

A case of tuberculosis was defined for the study as pulmonary using the case definition of NIDR, i.e. as a culture finding for *M. tuberculosis *in sputum or bronchoalveolar lavage (BAL), or as a culture finding for *M. tuberculosis *from another sample type in a case with sputum smear positive for acid fast bacilli. With this definition, 737 (70 % of the whole cohort) cases with pulmonary tuberculosis were identified. Species identification for Mycobacterium tuberculosis was carried out in every case. There were altogether 660 isolations of mycobacteria other than M. tuberculosis during the study period. Only culture positive cases were taken into the study in order to be able to study only the fully confirmed cases.

**Figure 1:**
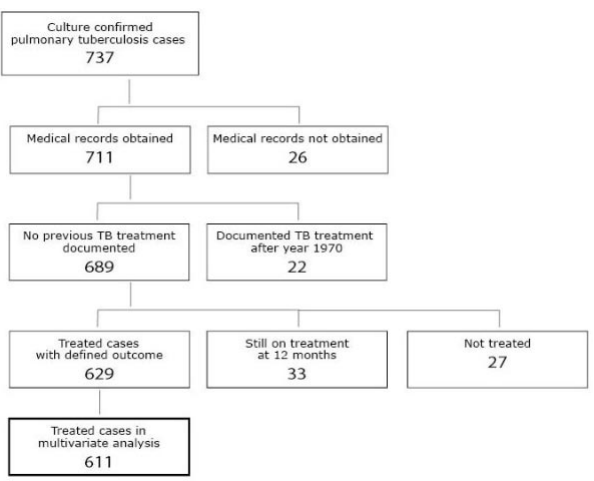
Composition of the national study cohort of culture-confirmed pulmonary tuberculosis cases treated at least 24 hours included in analysis.

Out of the 737 pulmonary tuberculosis cases, complete medical records were available from 711 (96%). Among these 711 cases, twenty-two (3.1%) had previously been treated for tuberculosis after the year 1970, and were excluded from the outcome analysis as re-treatment cases. Of the 689 cases, 33 were still on treatment at 12 months, and were also excluded from the analysis. Of the remaining 656 cases, 27 were not treated, among them 19 (70.4%) were men and 8 (29.6%) women. Of this group, with a median age of 82.0 years, 24 died before treatment and 3 were left untreated. Those without treatment were excluded from the main analysis of 629 actually treated cases (Figure 1).

### Definitions of treatment

Tuberculosis treatment was initiated in and supervised by the pulmonary departments of public hospitals in the great majority of cases. Chemotherapy actually given to each patient, according to record review, was grouped into six categories (Table 1). Definitions for the grouping were based on the national recommendations in Finland following the recommendations by WHO, ATS and BTS [27-32]. We have previously described the treatment grouping in detail [20]. Duration of treatment was assessed only for standard treatment groups (A – D). In the combination of isoniazid, rifampicin and pyrazinamide (HRZ) with or without an extra drug, the recommended duration of treatment was defined as 167 – 213 days. For the combination isoniazid, rifampicin and ethambutol (HRE) or isoniazid, rifampicin and streptomycin (HRS) ± extra drug, the recommended duration of treatment was defined as 243 – 304 days. Pauses of chemotherapy were recorded only when lasting at least one week, and calculated only for standard treatments.

**Table 1:** Definitions used in describing the treatment given to the cases in a national cohort of patients with pulmonary tuberculosis

**Treatment group**	**Drugs used in intensive phase**	**Duration of intensive phase [days]**	**Drugs used in continuation phase**	**Total duration of treatment [months]**
Standard treatment A	isoniazid + rifampicin + pyrazinamide	At least 54	isoniazid + rifampicin	At least 5 1/2
Standard treatment B	isoniazid + rifampicin + ethambutol or streptomycin	At least 54	isoniazid + rifampicin	At least 8
Standard treatment with short intensive phase C	isoniazid + rifampicin + pyrazinamide or ethambutol or streptomycin	Less than 54	isoniazid + rifampicin	At least 5 1/2 for A At least 8 for B
Standard treatment D	≥ 4 tuberculosis drugs, including the drugs in Standard treatment A or B	At least 54	isoniazid + rifampicin ± any other anti-tuberculosis drug(s)	At least 5 1/2 for A At least 8 for B
Other combination of tuberculosis drugs	Non-standard combinations of tuberculosis drugs, excluding the combinations above	NA^2^	Any combination of antituberculosis drugs	NA
Ineffective treatment	One antituberculosis drug used alone or in combination with a drug with limited antituberculosis activity^1^	NA	NA	NA

^1^E.g. fluoroquinolones

^2^NA = not applicable

### Definitions of outcome

The categories of WHO/EuroTB recommendation [6] for outcome monitoring are cure, treatment completed, failure, death, treatment interrupted (default), transfer out, and on treatment at 12 months. The duration of the follow up period is defined as 12 months from the beginning of the treatment or the date of diagnosis, and the first outcome registered as final.

The WHO/EuroTB category 'treatment interrupted' includes all treatment courses which remained shorter than defined above, whether caused by a patient or by a treating physician. For analysing these two separately, we divided 'treatment interrupted' into 'physician's decision to stop early' and 'default' for interruptions due to patient only [20]. The outcome was recorded as 'death', if the case died before starting the treatment (not included in the current analysis), during the treatment, or the date of death was within 14 days after cessation of the anti-TB drugs.

The outcome was categorized as favourable in cases of cure and treatment completed, and as unfavourable in cases of failure, death, default, physician's decision to stop early and transfer out.

### Definitions of origin, social and medical risk factors

A case was defined as immigrant if the country of birth was not Finland or, in the absence of country of birth, the nationality was other than Finnish. Immunosuppressive treatment was defined as corticosteroid treatment (>40 mg per day of any duration, or any daily dose with duration exceeding one month), cytotoxic or cyclosporine treatment, or radiation therapy during the preceding year. For inclusion in the group of social risk factors, a case should have a history of alcohol abuse, unemployment, imprisonment or homelessness recorded in patient records. Diabetes was defined as juvenile or adult onset disease on medication.

### Definitions of characteristics of treatment system

Specialty responsible for treatment was that of the unit treating patient as an inpatient or outpatient. Change of specialty included any change from one speciality to another during treatment.

### Ethical review

The ethics approval for this study was acquired from the National Research and Development Centre for Welfare and Health.

### Statistical methods

We used multinomial logistic regression model to assess the relationships between all potential predictors, listed in the univariate analysis, and a 3-class outcome variable, in which reference class was favourable outcome. To the final model significant predictors were selected using forward stepwise methods. Any variable whose univariable test had a p-value <0.20 was included in the multivariable analysis. P-values under 5% were considered as significant. For univariate results Chi squared test and Fisher's exact test were used.

## Results

In the study cohort of 629 cases, 386 (61.4%) were men and 243 (38.6%) women. The mean age was 62.9 years, the median 67.2. The proportion of cases aged ≥65 years was 339 (53.9%). The proportion of immigrants was 4.1% (26 cases), mainly from developing countries (Table 2). Only two patients in the cohort had HIV-coinfection. There were no prisoners.

**Table 2:** Univariate analysis of the association of patient – related characteristics with an unfavourable outcome in 629 cases treated for at least 24 hours

Variable		Total	Death			Other unfavourable		
		
		N	N	OR (95% CI)	p	N	OR (95% CI)	p
Sex	female	243	35	1		30	1	
	male	386	73	1.41 (0.90–2.204)	0.15	50	1.13 (0.69–1.84)	0.71
Age group	0 – 44	118	6	1		20	1	
	45 – 64	172	18	2.12 (0.81–5.56)	0.12	24	0.85 (0.44–1.63)	0.62
	65 -	339	84	5.89 (2.48–13.94)	**<0.001**	36	0.75 (0.42–1.38)	0.36
TB history	no	570	101	1		76	1	
	yes	59	7	0.57 (0.25–1.29)	0.21	4	0.43 (0.15–1.23)	0.15
Social risk factor^1^	no	330	76	1		33	1	
	yes	295	32	0.43 (0.27–0.68)	**<0.001**	46	1.42 (0.87–2.30)	0.18
Birthplace or nationality	Finland	603	107	1		72	1	
	abroad	26	1	0.23 (0.031–1.77)	0.22	8	2.77 (1.15–6.66)	**0.040**
Immunosuppression	no	548	78	1		76	1	
	yes	81	30	3.22 (1.92–5.42)	**<0.001**	4	0.44 (0.15–11.3)	0.15
Malignancy^2^	no	613	102	1		77	1	
	yes	15	6	4.26 (1.35–13.46)	**0.017**	3	2.82 (0.69–11.5)	0.15
Diabetes	no	537	86	1		73	1	
	yes	92	22	1.54 (0.86–2.63)	0.14	7	0.58 (0.25–1.31)	0.21

^1^Information missing for four cases

^2^Information missing for one case

Significant p-values are darkened.

A favourable outcome was achieved in 441 (70.1%) of the cases, consisting of those cured 199 (31.6%) and treatment completed 242 (38.5%). There were no treatment failures in the cohort. The proportion of cases defaulting or transferring out was 32 (5.1 %). For 48 (7.6%) cases, treatment was stopped prematurely by physician. Death was the outcome in 17.2 % (108/629) cases.

In univariate analysis, patient-related risk factors which were significantly associated (p < 0.05) with death, were age ≥65 years, social risk factor, immunosuppression and malignancy (Table 2). Treatment system -related risk factors which were significantly associated with death were the specialty of the treating unit (internal medicine, general medicine in primary care), less than five treated cases per year per unit, ineffective treatment combination and change in the treatment group (Table 3). The only statistically significant patient-related personal risk factor association for other unfavourable outcome (i.e. transfer out, default or physician's decision to stop early), was immigration (Table 2). Treatment system -related significant risk factors for other unfavourable outcome were internal medicine as the last treating specialty, standard treatment B and pause(s) during treatment (Table 3). For all unfavourable outcomes together, i.e. death and other unfavourable outcome combined, significant predictors in univariate analysis were gender, immunosuppression, malignancy, earlier TB, any other than a pulmonary unit being responsible at the end of the treatment, treatment group, pause(s) during treatment, less than five treated cases per year per unit and any change of specialty responsible for treatment.

**Table 3:** Univariate analysis of the association of treatment system – related characteristics with an unfavourable outcome in 629 cases treated for at least 24 hours

Variable		Total	Death			Other unfavourable		
		N	N	OR (95% CI)	p	N	OR (95% CI)	p

Specialty responsible for starting treatment^1^	pulmonary	579	90	1		76	1	
	internal medicine	34	11	2.52 (1.17–5.45)	**0.018**	3	0.82 (0.24–2.81)	0.75
	general medicine	6	2	3.06 (0.50–18.58)	0.22	61	1.81 (0.18–17.65)	0.61
	other	9	4	3.67 (0.97–13.34)	0.056	0	-	-
Specialty responsible for ending treatment^2^	pulmonary	531	61	1		67	1	
	internal medicine	28	17	18.72 (7.10–49.23)	**<0.001**	5	5.01 (1.49–16.89)	**0.009**
	general medicine	47	22	6.92 (3.59–13.34)	**<0.001**	4	1.15 (0.38–3.44)	0.81
	other	15	6	4.96 (1.66–14.77)	**0.004**	1	0.75 (0.093–6.11)	0.79
Change of specialty responsible for treatment^2^	no	530	66	1		71	1	
	yes	91	40	5.29 (3.21–8.72)	**<0.001**	6	0.74 (0.30–1.79)	0.68
Number of cases per year for unit giving initial treatment	1 – 4	36	6	0.91 (0.355–2.34)	0.85	5	1.19 (0.59–2.40)	0.64
	5 – 10	30	46	1.20 (0.46–3.16)	0.71	5	0.67 (0.32–1.43)	0.30
	11 – 29	286	50	0.84 (0.54–1.32)	0.46	33	1.16 (0.59–2.28)	0.67
	30 -	277	50	1		37	1	
Number of cases per year for unit responsible for ending treatment	1 – 4	93	33	3.27 (1.83–5.84)	**<0.001**	15	1.79 (0.89–3.63)	0.11
	5 – 10	38	5	0.80 (0.287–2.207)	0.67	5	0.96 (0.34–2.69)	0.39
	11 – 29	278	35	0.74 (0.44–1.23)	0.24	31	0.79 (0.46–1.36)	0.39
	30 -	220	35	1		29	1	
Treatment group	standard treatment A	309	64	1		25	1	
	standard treatment B	54	7	0.86 (0.36–2.06)	0.73	19	5.97 (2.92–12.20)	**<0.001**
	standard treatment C	33	1	0.13 (0.018–0.99)	0.05	6	2.03 (0.76–5.41)	0.16
	standard treatment D	155	24	0.72 (0.43–1.21)	0.28	16	1.22 (0.63–2.38)	0.55
	other combination	9	4	0.56 (0.25–1.25)	0.16	2	5.87 (0.94–36.81)	0.06
	ineffective	69	8	4.58 (1.00–21.01)	0.05	12	2.16 (1.01–4.58)	0.05
Treatment combination	standard (A – D)	548	97	1		66	1	
	non-standard	81	11	0.78 (0.38–1.55)	0.62	14	1.46 (0.77–2.77)	0.28
Change in treatment group	no	433	91	1		41	1	
	yes	196	17	0.40 (0.23–0.70)	**0.001**	39	2.05 (1.26–3.31)	**0.004**
Pause of treatment^2^	no	464	88	1		37	1	
	yes	157	16	0.64 (0.36–1.13)	0.14	42	3.97 (2.42–6.562)	**<0.001**
Pause during intensive phase^2^	no	483	91	1		44	1	
	yes	134	13	0.58 (0.31–1.082)	0.09	35	3.22 (1.95–5.32)	**<0.001**
Pause during intensive phase, due to side effect	no	523	97	1		56	1	
	yes	106	11	0.59 (0.30–1.16)	0.13	24	2.23 (1.30–3.84)	**0.005**
Pause during continuation phase	no	481	51	1		50	1	
	yes	70	8	1.49 (0.66–3.36)	0.35	22	4.18 (2.299–7.601)	**<0.001**
	NA (other + ineffective)	78						

^1^Information missing for one case

^2^Information missing for 7 – 12 cases

Significant p-values are darkened.

Significant independent risk factors for death in multinomial logistic regression model were male sex, high age, immunosuppression and any other than a pulmonary specialty being responsible at the end of the treatment. History of previous tuberculosis was inversely associated with the risk of death (p = 0.044). For other unfavourable treatment outcomes, significant risk factors were pause(s) in treatment, treatment group B and internal medicine being responsible at the end of the treatment (Table 4).

**Table 4:** Multivariate analysis of 611 cases (18 were left out due to missing values) treated at least 24 hours, odds ratio for death or other unfavourable (transfer out, default, physician's decision to stop early) outcomes. Reference category is favourable treatment outcome

Variable		Death			Other unfavourable		
		
		N	OR (95% CI)	p	N	OR (95% CI)	p
Sex	female	34	1		28	1	
	male	68	2.51 (1.42–4.45)	**0.002**	48	1.47 (0.83–2.61)	0.18
Age at diagnosis	risk per five years	102	1.29 (1.16–1.42)	**<0.001**	76	0.96 (0.88–1.041)	0.29
Immunosuppression	no	74	1		72	1	
	yes	28	2.11 (1.12–3.97)	**0.020**	4	0.32 (0.097–1.062)	0.063
TB history	no	96	1		73	1	
	yes	6	0.36 (0.133–0.97)	**0.044**	3	0.32 (0.087–1.16)	0.082
Specialty responsible for ending treatment	pulmonary	58	1		67	1	
	internal medicine	16	14.08 (4.76–41.66)	**<0.001**	4	6.89 (1.66–28.59)	**0.008**
	general medicine	22	6.24 (3.00–12.98)	**<0.001**	4	2.11 (0.65–6.86)	0.21
	other	6	4.72 (1.46–15.33)	**0.010**	1	0.58 (0.060–5.68)	0.64
Pause of treatment	no	86	1		36	1	
	yes	16	0.69 (0.33–1.47)	0.34	40	3.46 (1.92–6.27)	**<0.001**
Treatment group	standard treatment A	60	1		23	1	
	standard treatment B	7	0.85 (0.30–2.42)	0.76	19	5.92 (2.61–13.44)	**<0.001**
	standard treatment C	1	0.146 (0.017–1.22)	0.076	6	1.41 (0.48–4.12)	0.53
	standard treatment D	24	1.42 (0.76–2.67)	0.28	15	0.94 (0.45–1.95)	0.86
	other combination	8	1.15 (0.43–3.07)	0.79	11	1.54 (0.63–3.75)	0.34
	ineffective	2	2.64 (0.29–24.38)	0.39	2	3.54 (0.49–25.30)	0.21

Significant p-values are darkened.

For death and other unfavourable outcomes together, significant risk factors in the multinomial logistic regression model were male sex (p = 0.005), high age (p = 0.03), pause(s) in treatment (p < 0.001), treatment group other than A (p = 0.003) and any other than a pulmonary specialty being responsible for ending the treatment (p < 0.001). Earlier TB was a significant factor for favourable outcome (p = 0.012).

When we analysed all 656 cases in the cohort with known outcome, including those 27 without treatment, the associations observed in univariate and multivariate analysis as significant were the same as with the 629 cases of the presented analysis.

## Discussion

We analysed a large 2-year national cohort of culture-proven pulmonary tuberculosis cases for patient- and treatment-system related risk factors of unfavourable outcome. Apart from previously known patient-related risk factors for an unfavourable outcome, we observed a significant association with unfavourable outcome for the specialty responsible for treatment being other than pulmonary, but not for the volume of cases that the unit treated per year.

Due to the strictly controlled data collection process and high coverage, as reported previously [26], the data is highly representative. TB treatment recommendations and treatment organisation, as well as the proportion of foreign born, the age distribution and the case fatality rate have all remained unchanged since the study period in 1995–1996, making the analysis and conclusions valid for the present.

Due to relatively small number of deaths, the power of the study to investigate death as outcome was limited. The study was retrospective. Therefore, despite the meticulous conduct, it is possible that not all factors such as co-morbid conditions were recorded in the case notes.

Due to the fact that the TB patients in Finland are exceptionally old [13], contributing to the risk of death in several ways, we analysed separately the risk factors associated with death as outcome, as well as the risk factors for other unfavourable outcomes which may better reflect system features that may be amenable for improvement. Increasing age was strongly associated with death, contributing to the high case fatality ratio observed, and the major reason for not reaching the WHO targets for favourable outcomes. High age has been previously reported to be a risk factor for death, partly due to increasing comorbidities as well as the general physiological deterioration with age [7,12,21,22], because of which close monitoring of treatment in older patients is necessary. In older population it is often difficult to determine the causal relationship between tuberculosis and death. Based on the findings of an earlier Finnish study, it is probable that about a third of the deaths in our study were not directly attributable to tuberculosis [33].

In our aging population of TB cases, in the absence of TB/HIV co-infections, we found immunosuppression, virtually all due to other causes than HIV infection, from concomitant diseases and their medical treatment to be a risk factor for death, as has been previously reported [7,14]. For TB deaths in persons with diseases, which are themselves immunosuppressive and/or require immunosuppressive treatment, it is frequently difficult to explicitly determine the causal relationship between tuberculosis and death. As the clinical presentation of the underlying disease and TB may be difficult to differentiate, systematic use of rapid microbiological diagnosis and the early use of empiric treatment may be particularly valuable to improve outcome in this patient group.

In the analysis of treatment system -related factors, we found in univariate analysis significant or patterns of close to significant associations to death with specialty other than pulmonary beginning or ending the treatment, and with a switch in the specialty responsible for treatment. However, in multivariable analysis only the specialty service responsible for cessation of treatment being other than pulmonary was associated with death. Even though our data allowed controlling for a range of comorbid states, it is possible that in a patient population where half of the cases are older than 65 years, there could be more comorbidity in the group that ends up being treated in internal medicine and geriatric services.

An interesting finding in the univariate analysis for system-related risk factors was a reverse association of death with the number of cases treated per year by the unit in charge of ending the treatment. However, this association did not remain an independent predictor in multivariable analysis. For other unfavourable outcomes, there was no suggestion from univariate or multivariate analysis of an association with number of patient treated per year. While the volume of TB cases seen by each treating unit is becoming small, it is relevant to observe that favourable outcomes have been associated with increasing number of procedures for some other medical interventions such as treatment of leukaemia, AIDS and myocardial infarctions [23-25]. Pulmonary medicine in Finland treats the majority of tuberculosis cases, whether pulmonary or extra-pulmonary. The experience in the treatment of tuberculosis may therefore be greater in most pulmonary units than other specialty services. However, the volume of cases treated was used as a separate parameter in analysis. On the other hand, it is possible that the patient records do not fully cover all relevant comorbidities, which may be more common in other services than pulmonary. That in part can also explain association of death with the specialty service responsible for cessation of treatment being other than pulmonary. Overall, our results do not suggest that outcomes would improve by concentrating treatment in fewer clinical units in low incidence countries. This finding is relevant for low incidence countries with relatively little TB associated with immigration, homelessness, drug use and imprisonment, but may not be applicable in other settings.

Pause(s) in treatment were not found to be a risk factor for death. This can be due to deaths concentrating in the beginning of the treatment period in our study, leaving less possibility for pauses. On the other hand, pauses of treatment were a risk factor for other unfavourable outcome both in univariate analysis and in multivariate analysis. Pauses can be caused by side effects of treatment as well as non-compliance of a patient. There were more pauses in the initiation phase than in the continuation phase. Pauses during initiation may be more disadvantageous than in the later phase.

The potentially protective effect of history of earlier tuberculosis, although of borderline statistical significance, was an interesting finding. It seems likely that knowledge of tuberculosis in the past raises the early suspicion of the rare disease earlier, both in the patient as well as in treating doctor, leading to shorter delay in diagnostics and starting treatment, reported to affect outcome [34].

For unfavourable outcomes other than death, in univariate analysis association was found for being an immigrant, internal medicine service ending the treatment, belonging to treatment group B (commonly used when adverse effects necessitate switching of drugs or liver complications are anticipated), change in treatment group, and pauses in any stage of treatment. In multivariable analysis, however, only the system-related risk factors of internal medicine ending treatment, pause(s) and belonging to treatment group B remained independently associated with other unfavourable outcome. Earlier studies have found immigration as a significant risk factor [7,12,16]. For analysing immigration as a risk factor in our material, multinomial logistic regression model has a limited power, as the other unfavourable outcomes take place very early after start of treatment. Furthermore, the number of immigrants in this study is small, also limiting the power in multinomial model. No association between social risk factors and outcome was found, but that can depend on the dominance by unemployment rather than more extreme social issues which are rare in our country. In a recent analysis in London imprisonment, drug use and homelessness was found to be the most important predictors of poor outcome [35].

## Conclusion

A high proportion of deaths as TB treatment outcome are typical in low-prevalence countries [7,9,12,15-18]. Our results suggest that outcomes would not improve by concentrating treatment in fewer units with more patients. Poor outcomes associated with immunosuppression and advanced age, with frequent comorbidity, stress a low threshold of suspicion, availability of rapid diagnostics, and early empiric treatment as possible approaches in attempting to improve treatment outcomes in countries with very low incidence of TB.

## Competing interests

The author(s) declare that they have no competing interests.

## Authors' contributions

TV checked the data, led the analysis and interpretation of the data, and prepared an initial draft of the manuscript. PH and JO performed the statistical analyses and contributed to drafting the manuscript. KL contributed to designing the study, analysis and interpretation of the data, and to drafting the manuscript. MK led the collection of data and critically revised the manuscript. PR conceived of and coordinated the project, contributed to analysis and interpretation of the data, to drafting the manuscript, and critically revised the manuscript. All authors read and approved the final manuscript.

## Pre-publication history

The pre-publication history for this paper can be accessed here:


